# Is Open Science Neoliberal?

**DOI:** 10.1177/17456916221114835

**Published:** 2022-12-07

**Authors:** Duygu Uygun Tunç, Mehmet Necip Tunç, Ziya Batuhan Eper

**Affiliations:** 1 Department of Philosophy, Middle East Technical University; 2 Department of Social Psychology, Tilburg University; 3 Department of Political Science, Galatasaray University

**Keywords:** scientific reform, open science, neoliberalism, axiology, science policy, ideology

## Abstract

The scientific-reform movement, frequently referred to as open science, has the potential to substantially reshape the nature of the scientific activity. For this reason, its sociopolitical antecedents and consequences deserve serious scholarly attention. In a recently formed literature that professes to meet this need, it has been widely argued that the movement is neoliberal. However, for two reasons it is hard to justify this widescale attribution: First, the critics mistakenly represent the movement as a monolithic structure, and second, the critics’ arguments associating the movement with neoliberalism because of the movement’s (a) preferential focus on methodological issues, (b) underlying philosophy of science, and (c) allegedly promarket ideological proclivities reflected in the methodology and science-policy proposals do not hold under closer scrutiny. These shortcomings show a lack of sufficient engagement with the reform literature. What is needed is more nuanced accounts of the sociopolitical underpinnings of scientific reform. To address this need, we propose a model for the analysis of reform proposals, which represents scientific methodology, axiology, science policy, and ideology as interconnected but relatively distinct domains, and thus allows for recognizing the divergent tendencies in the movement and the uniqueness of particular proposals.

The poor replicability of empirical claims in the published literature of some scientific disciplines (Button et al., 2013; Camerer et al., 2016; Ioannidis, 2005; Open Science Collaboration, 2015) triggered a long process of reflective questioning in these areas. The observation, shared by many, that reproducibility-related problems are rooted in established but faulty research practices (Bakker et al., 2012; John et al., 2012; Simmons et al., 2011) has turned into a movement aiming to amend these practices (Spellman, 2015), which often goes by the name open science.^1^ Although envisioned reforms are mostly methodology-related, the long-term consequences of these modifications have the potential to lead to some very fundamental changes, such as transforming the culture and organizational structure of scientific practice (e.g., whether individuals or teams are funded), the science–society relationship (e.g., how scientific results are disseminated), and even the scientists themselves (e.g., rethinking the academic promotion criteria). Therefore, critical inquiries regarding the probable downstream sociopolitical consequences this movement might have on science, the academic establishment, and society are massively needed.

A number of such critical articles which examine the sociopolitical correlates of the scientific-reform movement have been written so far. Some of these strongly resonate in directing a very similar criticism against reform-minded meta-scientific theorizing, namely being market-oriented, antilabor, or neoliberal (Callard, 2022; Flis, 2019; Levin & Leonelli, 2017; Mirowski, 2018; Morawski, 2019; Peterson & Panofsky, 2020, 2021). Embedded in these criticisms is that the scientific-reform movement, at a deeper level, shares the assumptions or ideals of the neoliberal status quo, which creates the very problems that the reform movement purports to solve. Or even worse, it helps the status quo survive by reproducing the neoliberal ethos under new garments. We believe there are strong reasons to suspect that these analyses are both overly simplistic and broadly inaccurate, thus falling short of providing the needed critical sociopolitical reflection on the scientific-reform movement. In this article, we would like to throw a little critical light onto the critical analyses associating the scientific-reform movement with neoliberalism.^2^

## Common Ground

The scientific-reform movement has focused much of its efforts on methodological and statistical issues (Wiggins & Christopherson, 2019). Although from the beginning there have also been other concerns (such as the incentive structures in science or the prominent values of the contemporary scientific culture; see Giner-Sorolla, 2012; Ioannidis, 2005; Koole & Lakens, 2012; Nosek et al., 2012), these were brought to attention chiefly by virtue of their association with methodological-statistical concerns. Thus, a big part of the scientific-reform movement can be characterized by a critical attitude toward the questionable methodological habits in the social, behavioral, and life sciences; the effort to develop more rigorous justification standards that would increase the credibility of published literature; and the establishment of the infrastructure and platforms to support the application of these standards.

Critics find this almost exclusive focus on methodology in dealing with the replication crisis inadequate and mistaken. We agree with the argument that methodological concerns do not exist in a normative vacuum. There are not many “purely methodological” issues, which are not connected in any way to how one conceives the nature and aims of scientific inquiry and how these fit into the broader sociopolitical context of scientific practice. We also believe that the scientific-reform movement has had a relatively myopic vision of how the entrenched methodological habits in whole scientific fields can be broken and novel practices can be cultivated, which requires a deeper and realistic analysis of the social organization of science, the “scientific ideologies” that shape and maintain it, and the types of personal attitudes and behaviors it selects and weeds out. Such an analysis should go beyond delegating the responsibility to “incentive structures” and “research culture,” both of which can be argued to have become buzzwords that hardly have any meanings beyond indicating the obvious—that the problem is systemic. However, we must also note that there have been promising signs of significantly increased attention to the social dimension of science reform recently (e.g., Derksen, 2019; Higginson & Munafò, 2016; Lilienfeld, 2017; Moher et al., 2018; Schiavone et al., 2021; Smaldino & McElreath, 2016; Tiokhin, Panchanathan, et al., 2021; Tiokhin, Yan, & Morgan, 2021).

Thus, we are also largely in agreement with the critics that a deeper understanding of the social organization of science is still lacking in the scientific-reform movement. Yet we substantially diverge from these critics on three main accounts: the “extent” of the dependence of methodology on normative questions such as social values and political ideology; whether the science reformers’ preferential focus on methodological issues (at the expense of other acute or chronic problems in academia) promotes and reproduces the neoliberal status quo—the argument from omission; and whether the philosophy of science or the sociopolitical implications of scientific-reform proposals themselves reflect neoliberal tendencies—the argument from the foundations and the argument from the commission. We tackle these one by one.

## To What Extent Are Methodological Questions Dependent on Normative/Political Questions?

We start with the presumed inherent link between methodological and ideological stances in science. We think this is the best starting point for examining the arguments by these critics because even before looking at the veracity of the actual content (i.e., if the reform movement is neoliberal), two core methodological problems stand out: First, these analyses conjecture a strong determining influence of normative questions on methodological issues, and, second (partly because of that), they tend to picture scientific-reform proposals as belonging to a uniform, monolithic movement—which knowingly or unknowingly serves the neoliberal agenda. We believe there are good reasons to think that both assessments are incorrect, but let us first see what the critics’ arguments are.

For Mirowski (2018), methodological-reform proposals to increase the transparency of research processes reflect a deep distrust of individual scientists. The reason for this distrust, the author claimed, is that the reformers do not believe that individual scientists are able to “comprehend the amount of information” necessary for reaching well-justified inferences, and thus reformers think “experts (and scientists) should not be accorded much respect,” and they reduce experts “to the same epistemic plane as rank amateurs” (p. 188). So, supposedly what is concealed by the reformers behind the deceptive banner of increasing epistemic efficiency (i.e., improving scientific inference through openness and better policing of methodological decisions) is the neoliberal ideology trying to rob individuals of decision-making power and delegating it to “the markets.” Levin and Leonelli (2017), for example, maintained that open practices are not only methodological tools for assessing and increasing overall rigor (as professed by science reformers; see Chambers, 2017; Frankenhuis & Nettle, 2018; Wagenmakers et al., 2012) but also entail “articulations of what is valuable and what relationships exist to generate, ensure, and reinforce such value” (p. 284). The “value” in question, according to the authors, is governed mostly by the market-related issues in the reform literature. So, what drives the calls for openness first and foremost is financial-efficiency concerns rather than epistemological/methodological considerations. Furthermore, the authors argued that mandatory open-science practices would cause openness to be governed not by “localized principles of trust and gifting [but] instead . . . through generalized principles of economic value” (p. 297). Callard (2022), as another example, argued that similarities between “(experimental) replication and (social) reproduction processes” do not end in both involving repeating something (p. 199). According to the author, “if you are worried about reproducibility in psychology, you should be worried about current conditions undergirding the reproduction of labor in the university” (p. 200), and this is because the 2007–2008 financial crisis and its consequences for labor have had a considerable impact on the “emergence, persistence, and very shape of a crisis of replication” (p. 201). The most striking form the argument (i.e., strong determination of methodological debates by normative positions) takes is that the “crisis” of faulty methods is “invented” by the reform movement as a response to (or as an effort to address) a corresponding ideological crisis of neoliberalism. Mirowski (2018) proclaimed that the overt discourse about “replication failure, growing retractions and wonky statistics” is nothing but an effort to advertise a change in the academic business model, and to accomplish that change reformers are supposed to “evoke the magic of the marketplace to displace centuries-old practices of science” (p. 186). Voicing similar opinions that are mostly inspired by Mirowski (2018), Morawski (2019) argued that “the purported malaise” of established habits in science “might not be a substantive rupture . . . but, rather, an ideological backdrop for the open science movement to better establish a neoliberal market and social organization.” However, we must admit that we are not sure whether Morawski genuinely adhered to the “strong-determination” perspective because when it comes to reformers the author also pointed out a problematic disregard for “the distinct separation philosophers traditionally make between epistemic matters and social ones.”

## A Preliminary Model for Representing the Interrelations of Methodology, Axiology, Science Policy, and Ideology

**Fig. 1.**
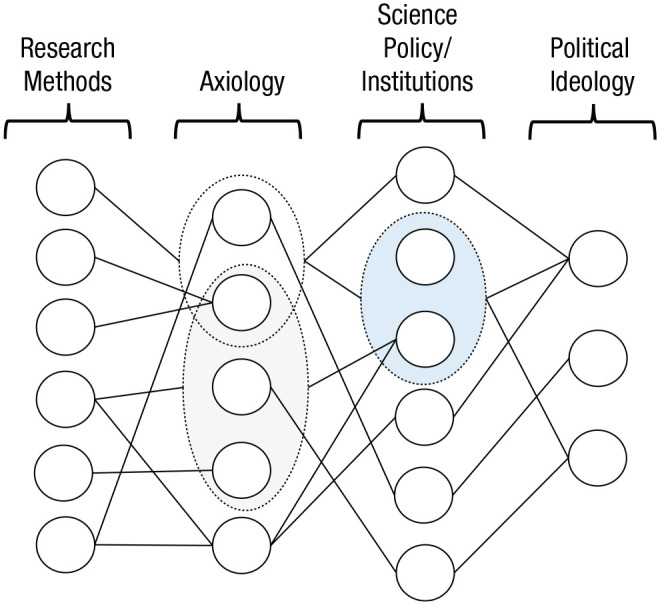
Complex network of interrelations between methods, axiology, science policy, and ideology.

We are of the opinion that not even the staunchest positivist would argue that methodology as practiced by scientists is always an insular domain. Thus, we do not suggest that it is impossible to draw some connections between methodological-reform proposals and particular normative belief systems such as ideologies. Yet we are skeptical about whether the link between methodological issues and ideological values (a) follows such an immediate route and (b) is as strong as the critics seem to assume. Instead of an immediate and strong link, we can at best speak of a relationship that is variously mediated—in the least by scientific axiology, scientific-policy choices, and the institutional structure of science. A preliminary outline of a model for such a relationship is shown in Figure 1.

Figure 1 illustrates the complex relationships between methodological norms and practices, the goals and values of scientific inquiry, scientific policies and the institutional structure of science, and broader sociopolitical values and ideas. This model is a descriptive one in regard to the nonhierarchical relationships it represents between individual elements belonging to these four domains. We believe that such a representation of the four relevant domains of meta-scientific analysis and their interrelations can serve as a useful reference point in analyzing a multifaceted phenomenon such as the scientific-reform. When evaluating any methodological reform proposal, it is worthwhile to analyze first which scientific goals or values it serves or conflicts with. This analysis will guide the consideration of which institutional structures or scientific policies could serve or hamper the realization of the identified scientific goals or values and what kind of possible downstream effects the proposal might have on the social and institutional organization of scientific activity in the longer term. In what follows, we elaborate on the nature of the relationships between these four domains of meta-scientific analysis as a basis for evaluating to what extent political questions can be said to have a bearing on methodology-focused reform discussions and vice versa.

Methodology comprises principles for evaluating the empirical support of scientific claims. These principles can be mathematical formulas for generating empirical statements (such as statistical-inference techniques) as well as general rules for data generation and evaluation (such as “data used in formulating a hypothesis should not be used to test the same hypothesis”). Axiology is the domain of scientific goals and values that we want scientific claims to achieve or manifest, such as explanation, prediction, novelty, observational accuracy, intersubjective testability, or generality. These values are not always complementary, so they can be in conflict, such as when the goal of explanation contradicts the goal of prediction. That is, axiology has a subjective element to it, and there can be no absolutely objective basis for preferring one over the other. Such values guide methodological preferences, and they can be invoked for comparing the success of several methodological practices. Most scientific values are multiply realizable; that is, they can be achieved or manifest via different methodological procedures. Moreover, certain methodological practices can be justified in reference to multiple scientific values; thus, scientists who subscribe to different philosophies of science may nonetheless use or refrain from the same methods. Figure 1 illustrates this through the multiplicity of the links between elements of methodology and axiology.

Scientific methodology is closely related to scientific axiology but does not have an immediate relationship to other domains of meta-scientific analysis. That is, the relationship between methodology and science policy/ideology is mediated by scientific axiology. Methodological discussions do not directly imply what practices should be positively evaluated, promoted, or disincentivized by scientific institutions. Rather, they address which methods are better suited for which scientific aims. Consequently, proponents of the same methodological perspectives might diverge in their perspectives on axiology and science policy, or advocates of the same policies might subscribe to incompatible methodological perspectives. It is also possible for a methodological discussion to have no relevance for science policy and the institutional structure of science, or for a discussion of institutional reform to have no methodological implication. Last, the broader sociopolitical dimension of science as an establishment is a feature of most scientific institutions and policies but has no direct, immediate relevance for axiological and methodological questions. Thus, proponents of the same methodological perspectives and even the same scientific goals could substantially diverge in their sociopolitical perspectives on the institutional organization of academia and which problems deserve to be addressed by science policy.

Thus, we can hardly speak of one-to-one relationships between elements of methodology, scientific axiology, values and norms in science policy and the institutional structure of science, and sociopolitical values. We can at best speak of relatively stronger or weaker attractions between elements of methodology, axiology, science policy, and ideology, which we can conceive in a way that is akin to what is called “elective affinities” in describing the natural attraction between individual and group-based dispositions and certain ideologies in political psychology (Jost et al., 2009; Tomkins, 1963; see also Weber, 2001).

We give two pertinent examples from the scientific-reform movement that testify to the complexity of the relationships between methodology, axiology, science policy/scientific institutions, and ideology. Our first example, preregistration, can illustrate how the same research method can be (a) mobilized for or (b) interpreted as endorsing different axiological perspectives. Then we discuss how these axiological perspectives can be associated with different science-policy proposals. Nosek et al. (2018) suggested that the main function of preregistration is to distinguish prediction from postdiction. According to these authors, preregistration is a preventive measure against various types of ad hoc reasoning (e.g., HARKing, *p*-hacking) that influence the observed outcomes in a study. From this perspective, the scientific value associated with preregistration is what philosophers of science call “use novelty” (Worrall, 1985, 1989). According to Lindsay et al. (2016), preregistration is a methodological tool for preventing exploratory investigations from passing themselves as confirmatory tests. “The problem” for these authors “is not data exploration” because it “can lead to new ideas and discoveries.” The real problem is that “exploration differs from planned hypothesis testing,” and “flexible analytic practices . . . dramatically increase the chances of erroneously rejecting null hypotheses (Type I errors)” and thus decrease the test’s accuracy. From this perspective, the scientific value associated with preregistration is what philosophers of science call “exactitude” (Reichenbach, 1961). According to Lakens (2019), preregistration is for allowing “others to transparently evaluate the capacity of a test to falsify a prediction” (p. 222). He further explicated that “[t]he severity with which a claim is tested is not necessarily impacted by preregistration” (p. 227) because, for example, the internal constraints can be known to the research team whereas others can never know whether they were there before the tests. So, this main function of providing others with information about the severity of a test, according to the author, must be distinguished from positive externalities such as counteracting bias (such as confusing prediction with postdiction), which can be achieved by means other than preregistration. From this perspective, the scientific value associated with preregistration is what philosophers of science call “falsifiability” (Popper, 1959).

To illustrate how these different axiological interpretations translate into concrete science-policy proposals, we can compare Nosek et al. (2018) with Lakens (2019) in terms of to what extent these perspectives are in consonance with “preregistration badges.” Open-science badges are given in recognition of open activities such as data sharing and preregistration and are thought by some to be a cost-effective way of incentivizing open science (Kidwell et al., 2016). From the axiological perspective of Nosek et al. (2018), it makes all the sense to use badges for preregistration because the existence of a preregistered analysis plan is a very good criterion for distinguishing use-novel hypothesis tests from others. Lakens (2019), on the other hand, did not see such a value in the mere existence of preregistration. He conceptualized it as a tool for allowing researchers to evaluate test severity. Without having access to the content of preregistration, it cannot be said whether and to what extent the hypothesis and its test display the value of falsifiability.

In our second example, we focus more on the axiology-science policy-ideology association as featured in our model. Established practices of peer-review have received serious criticism from the science reformers for failing to deliver scientific-error detection and quality control (e.g., Nosek & Bar-Anan, 2012; Wicherts et al., 2012). These “failings” of peer review are identified by almost all critics in reference to similar, compatible axiological concerns regarding quality control (see Jubb, 2016). But proposals for changes in the science policy and scientific institutions to remedy the shortcomings of the current peer-review system are extremely varied. We can mention in one breath peer-reviewed peer review (Wicherts et al., 2012), crowdsourced review (Ford, 2013), publish and then review (journals-as-curators model; Eisen et al., 2020), postpublication peer review (Kriegeskorte, 2012), the red-team approach (Lakens, 2020), and the 450 movement (Heathers, 2020). These different science-policy proposals present a very divergent array of assessments about current peer-review practices regarding both the sources and maintaining factors of the problem and the aspired future state of academic publication. This is exactly where ideological influences kick in. For example, the idea of peer-reviewed peer review (where open reviews are rated by other scholars postpublication) strongly resembles the web-rating sites that function mainly to protect consumers by allowing them to access a crowdsourced reputation metric. Therefore, it can be argued that the main aim of peer-reviewed peer review is to protect the “consumers” of scientific knowledge by introducing another layer of quality control. Crowdsourced review (and in some measure postpublication peer review) envisions a more communitarian solution, resonating strongly with the ideal of participatory democracy in science. The publish-then-review model aims to abolish the gatekeeping function of peer review that sometimes can take some form of censorship in the current model and conceive journal publication just as an additional source of credibility. Thus, the publish-then-review model can be said to adhere to a strong pro-free-speech stance. The red-team approach envisions that scientists make an agreement with independent experts to scrutinize their own research. The red-team market is an application of this approach in which experts are hired for their services. The red-team market is indeed a promarket solution to the problems of peer review and it is one of the few policy proposals coming from the reform movement that can somehow can be associated with platform capitalism. The 450 movement, another science-policy proposal about peer review, problematizes the uncompensated, invisible labor of reviewers and suggests sending academic journals (which make enormous profits each year; see Buranyi, 2017) a contract whenever they request a review, asking them to pay the due price of reviewers’ efforts. So, we think it is safe to say that it is a very prolabor proposal, bearing a resemblance to other grievances in the history of labor about unpaid work.

## Critics’ Model of Science Behind the Methodology-Ideology Association: Kuhnian Holism

As can also be seen in the preceding examples, the connection between political ideology and scientific methodology is open to various mediators and hence can be established in a myriad of ways. Consequently, it is close to meaningless to try to link any ideology such as neoliberalism to particular methodological perspectives. The critics of the scientific-reform movement apparently subscribe to a very antiquated vision of science in which scientific theories, methods, and scientific values are linked via invariable relationships—which Laudan (1986) called “holism”—so that they may (or may not) contribute to or be selected and promoted in toto by scientific institutions and policies. The holist view of science envisions inherent, not easily malleable relationships between the answers one may give to three questions: What (kind of) entities populate the world (i.e., ontology), what are the most suitable methods and tools for studying these entities (i.e., methodology), and what is the epistemic goal or ideal of such an inquiry (i.e., axiology; see also Laudan, 1986)? One of the most famous proponents of the holistic model of science was Kuhn (see Laudan, 1986, p. 68), so it might be reasonable to think that Kuhn or his intellectual legacy is the source for this holistic view of science that appears to be endorsed by the critics. Some of the critics of the scientific-reform movement also express that they find great potential in Kuhn in terms of a more realistic philosophy of science (see, e.g., Flis, 2019). Kuhn (1962) further inflated the content of the holistic model of science to the magnitude of a “paradigm,” beyond the (already vast) scope intended by the rationalist school of philosophy of science he directed his criticism to that also incorporated the broader social processes in which scientific inquiry is embedded. For Kuhn, the axiology of science is not restricted to epistemic values but to all kinds of normative attitudes that make up a worldview (or Weltanschauung). Moreover, in the idea of a paradigm, Kuhn strongly consolidated the questions of ontology, methodology, and axiology so that their interrelationship is no longer just a strong affinity on rational grounds but a necessary link—to the effect that all stand or fall together, and there can be no sufficiently rational reasons for their collective demise. If science is such a holistic structure, the influence of political ideology on the institutions and policies of science could be directly transmitted to methodology and thereby to scientific theories and even to scientific facts, and vice versa. However, as Laudan forcefully argued, the history of science offers plenty of examples that show that scientific ontologies, methodologies, and axiologies not at all stand or fall together. Scientific change happens in a much more piecemeal fashion—sometimes simply in the form of stand-alone methodological advancements. This holist picture, which the critics presumably take over from Kuhn, is extremely simplistic and thus has very limited, if any, explanatory power that can serve as a critical investigation of the scientific-reform movement.

## The Reform Movement Is Not a Monolith

As a consequence of the complexity of the interrelations between various domains in which science reform can have repercussions, the reform movement is not a monolith.^3^ Because there are various methodological and axiological strands, there is also substantial diversity in proposals concerning science-policy and institutional reform. The reform movement hosts too many different axiological/political tendencies to be summarized or characterized by a single adjective. Thus, there is not an “ideology of the scientific-reform movement.”

We would also like to add to this discussion that although the movement is ideologically diverse at the moment, it may evolve into a more monolithic structure. Even if this were to become the case, methodological issues would keep their relative independence from policy and ideology. Although methodological norms in social sciences often have downstream repercussions in terms of science policy and ideology, it would be a rather radical position to argue that the epistemological value of scientific outputs using a set of methodological norms solely depends on its associated ideology or sociopolitical virtues/vices. That is, epistemological axiology is not solely dependent on political axiology. A neoliberal open science might lead to quite detrimental consequences such as compounding the disadvantages associated with existing societal inequities (Ross-Hellauer, 2022). However, even under the scenario in which open science is completely subsumed by its neoliberal strains and ceases to aim for more equity, the spreading of methodological reforms (such as registered reports; see Chambers et al., 2015) can still increase the credibility of published scientific findings compared with the current status quo which is rife with publication bias (Fanelli, 2010). There is little reason to think that reformist policies should be utterly neutral in terms of their ideological/political antecedents and consequences to be effective in increasing the veritic value of a scientific literature.

That, of course, does not mean that the reformers’ diagnoses of various kinds of problems of science and possible remedies would not be enriched by more attention to the potential downstream consequences of methodological reform on the sociopolitical organization of the scientific establishment. However, to play such a role, a critical analysis of the scientific-reform movement should put forward a sophisticated examination of the complex interrelations between the four domains we specified, namely between particular methodologies, axiological perspectives, science-policy proposals, and broader sociopolitical ideas—not only in relation to the question of neoliberal bias but as a general methodological requirement. Across-the-board generalizations which do not recognize the relative independence of these different domains simply do not fit the complexity of the matter. Therefore, we think that the question “Is science reform market-oriented/antilabor/neoliberal?” is formally wrong. But this analysis should not be rejected only on a priori grounds because it might nonetheless contain true premises that can advance the meta-discussion on scientific reform. Thus, let us examine next why critics choose to characterize the reform with these labels.

### How do critics define neoliberalism?

The term “neoliberalism” has been defined variously in the past, and it may not be easy to settle on a conclusive definition (see Ferguson, 2010; Ganti, 2014; Venugopal, 2015). But many people, including Mirowski, who was one of the first authors to associate this label with the scientific-reform movement, see as the telltale of neoliberalism the attitude of invoking unbridled market forces in tackling problems for which no such solution is appropriate. So, according to this reading, in the background of the neoliberal perspective, there is the idea that introducing regulation into self-regulating systems (the paradigmatic case being unregulated markets) reduces their efficiency. Neoliberalism, therefore, is seen as “laissez-faire” taken to its extreme—an approach that does not limit noninterventionism to economical matters but considers it the best way to “organize” all areas of social life. Mirowski regarded neoliberalism not only as a socioeconomic/ideological stance but also as an epistemic attitude that pervades many other domains (Mirowski, 2009).

### Why is reform neoliberal? The argument from omission

The critical analyses which argue that methodological-reform proposals reflect market-oriented or neoliberal sociopolitical values do not necessarily attribute an intentional stance to the reformers. Some critics invoke what we call “argument from omission,” in which science reform is deemed neoliberal either because of its disproportionate attention to methodological and statistical issues or because it ignores the far-reaching promarket and antilabor consequences of its methodological proposals on science policy. Critics find these omissions quite meaningful. Callard (2022) maintained, for instance, that it is “unsurprisingly” difficult to “discern questions of labor—and exploitation, and appropriation—within the replication crisis” (p. 203) and the bearing of the labor conditions because of the 2007–2008 financial crisis “on the emergence, persistence, and very shape of a ‘crisis of replication’” (p. 201). Questions of labor are invisible not only in the reformers’ diagnoses of a crisis, according to Callard, but also in their proposed remedies: The reform proposals reflect “a fantasy of work without workers” (p. 204) because open-science practices require additional labor but nobody in the movement, the author claimed, discusses who is going to bear the burden of this extra work.

However, many in the movement indeed suggested that it would not be possible for the reform to take hold if work related to open science is not compensated (Munafò et al., 2017; Nosek et al., 2015). It can also be argued that the entire discussion regarding incentive structures in science aims to get invisible methodological groundwork rewarded (see, e.g., Tiokhin, Panchanathan, et al., 2021). Furthermore, the recently introduced Recognition & Rewards (2021) program in the Netherlands is an actual policy change that explicitly associates itself with open science and acknowledges and compensates such non-visible work. (Dutch Research Council, 2021). Considering all of these steps towards reforming the labor conditions in science, we cannot see how reform proposals envision “a fantasy of work without workers.”

Of course, faulty methodological habits (i.e., the primary focus of the reform movement) do not exhaust all of the problems that pervade academia today. One instantly thinks of institutional racism, classism, and sexism; the increasing precarity of academic labor; and public distrust in academia as other prevalent issues. Methodological problems may even not be the most pressing ones. However, it is also not at all odd that the attention of scientists would often be directed at the methodological dimension of science because improving scientific-justification practices is an essential part of scientific inquiry as well as a core responsibility of scientists toward their peers and the public. Moreover, science reform, however conceived, cannot and should not be treated as snake oil that cures all of these ailments. It is utterly inexpedient to expect that a movement that aims to improve the quality of scientific inferences should also solve all the systemic problems of the scientific establishment. It would also do injustice to the seriousness of the issues of this kind to be casually put in the same basket as problems of a much more technical nature that are suitable to be addressed by policies of a much more specific scope and vision.

### Why is reform neoliberal? The argument from the philosophical foundations

Another line of argument presumes a deeper connection between reform and neoliberalism based on an assumption that reform and neoliberalism share a common intellectual root: Sir Karl Raimund Popper. This group of critics presumes an intrinsic connection between the Popperian philosophy of science and neoliberalism. The claim, thus, is that any methodological reform or science-policy proposal that has been inspired by any strain of Popperian falsificationism is by nature neoliberal. It has been observed by many (Derksen, 2019; LeBel et al., 2017), including the critics (Davidson, 2021; Flis, 2019; Mirowski, 2018), that the reform movement is heavily inspired by the hypothetico-deductive school and thinkers such as Popper, Lakatos, Meehl, and the like. However, what is absent in the critics’ arguments is the theoretical justification concerning how Popper’s philosophy of science entails neoliberalism. Mirowski (2018), for instance, wrote that “in the process of attempting to square this circle, many of the prophets of open science unselfconsciously cite Friedrich Hayek and Karl Popper, two early members of the Mont Pelèrin Society” (p. 193). Elsewhere, Mirowski portrayed the Mont Pelèrin Society to be the intellectual birthplace of neoliberalism (Mirowski, 2009). We think that, if Popper’s political beliefs, society memberships, or personal relationships are to be linked to his philosophy of science, it should be explained in detail how these contingencies influenced his ideas in that domain. Similar ascriptions (i.e., neoliberal, libertarian) are also made for Paul Meehl (while emphasizing his influence on the reform literature), yet any explanation of how these alleged political tendencies affected Meehl’s ideas on scientific methodology is also missing (see Davidson, 2021). Flis (2019) attempted to explain why reformers’ philosophy of science necessarily renders the movement promarket. Flis suggested a link between “reformers’ model of science as a system” and their alleged belief in the “hidden hand of the market” and associated this link with the observation that the reformers still subscribe to a “post-positivist or logical positivist philosophy of science” and “different articulations of Popper’s falsificationism or the hypothetico-deductive model” (p. 175). However, we cannot really say that we understand why the author thinks modeling (or marketing) science as systems should necessarily imply that the models are envisioned as “self-regulating.” We could actually speculate a model transfer from economy to science in the idea of a self-regulating system. Ironically, however, this idea can be linked to the idea of a self-correcting science, which arguably was a common illusion that shattered for many with the outbreak of the replication crisis. A widely shared attitude in the reform movement is that science does not correct itself unless scientists actively do so. As can be seen in Vazire and Holcombe (2022), the reform movement positions itself vehemently against that “self-regulatory system” model of science. In sum, although it might be possible to associate reformers’ philosophy of science with certain promarket or neoliberal ideological stances, critics still have not been able to provide sufficient justification for that argument.

### Why is reform neoliberal? The argument from commission

A concrete application of the neoliberal perspective to the domain of science might be the belief that when organized in the form of deregulated markets, scientific fields are capable of maximizing and maintaining research quality. The exact meaning of deregulation might change depending on the particular domain. Thus, when the critics associate increasing the precarity of academic labor (Callard, 2022), regard of economical concerns as the ultimate source of value (Levin & Leonelli, 2017; Peterson & Panofsky, 2021), dehumanizing scientific criticism by automatizing quality control with the help of computer technology (Peterson & Panofsky, 2020), or endorsement of platform capitalism (Mirowski, 2018) with the scientific-reform movement, they see an underlying tendency for neoliberal deregulation. Callard posited that “the epistemic constraints and norms that govern psychological research are themselves attached to particular kinds of social formations” that are “shaped through diverse kinds of labor relations” (p. 202). Callard further implied, in reference to Morawski’s work on the reformers’ representation of the scientific self, that the researcher and the research culture in reform literature reflect a “neo-classical” economic framework. Callard argued that the virtues of “transparency, openness, and efficiency” championed by the scientific-reform movement appear “in university ecologies that have embraced New Public Management” (p. 204)—the par-excellence reflection of neoliberalism in the university. Callard claimed that the effort to increase transparency, openness, and efficiency “exacerbate problems for labor” by installing novel means for appropriating uncredited and uncompensated (thus “invisible”) labor, creating additional burdens on the already precarious researchers, leading to new forms of authority and exploitation, and doing so “in highly unequal, gendered and racialized ways” (p. 204). Levin and Leonelli (2017) similarly asserted that “the modern instantiations of openness that permeate the open science movement are intertwined with particular political-economic regimes, such as the increasing commercialization . . . and the exercise of proprietary intellectual property regimes” (p. 283-4). Thus, the movement’s “focus on freedom, democracy, individualism, and free competition is not necessarily opposed to the proprietary and corporate” and is quite compatible with the neoliberal mindset. This is because openness, as cherished by the reformers, involves “circuits of exchange,” and they “generate, ensure, and reinforce such” economically defined value (p. 284). Peterson and Panofsky (2021) maintained that the economism at the heart of meta-science results in the adoption of efficiency as the primary value of scientific reform, and the reformers promote liberal values such as transparency and accountability to increase efficiency. In another work, Peterson and Panofsky (2020) interpreted the reformers’ conception of efficiency as the removal of “friction” that slows down science by novel technological means. They claimed that this is a familiar blueprint in the neoliberal market ethos, namely the usage of “advances in technology to innovate and transform stagnant industries” (p. 18). This is accompanied by what they presented as the reformers’ philosophy of science, a “post-human theory of science in which the limitations of human bias can only be overcome with total transparency and machine intervention,” and the reformers’ image of the scientists as “in the business of producing, curating, and maintaining data” (p. 20). They further argued that the science reformers can imagine the task of improving the methodological quality of science as a “doable problem” only because of their “quantitative, atheoretical, positivist” (p. 24) meta-science that turns scientific activities “into objects for enumeration, measurement, comparison, evaluation, and, ultimately, manipulation” (p. 27). Mirowski (2018) was most explicit in his characterization of the ideological underpinnings of the scientific-reform movement: “The open science movement is an artifact of the current neoliberal regime of science, one that reconfigures both the institutions and the nature of knowledge so as to better conform to market imperatives” (p. 172). Mirowski went even further by arguing that “the [open-science] agenda is effectively to re-engineer science along the lines of platform capitalism, under the misleading banner of opening up science to the masses” (p. 171). He underlined the “paramount importance” of exposing this underlying reality also because “many scientists are attracted to the open science movement because they believe it to be a renunciation of older commercial models” (p. 187).

The encompassing narrative in these criticisms is that the scientific-reform movement imagines scientific production in a way that is akin to commercial production. The movement envisages improving its efficiency by rendering scientific inquiry transparent and open to automatized scrutiny by technological means and ignores the humanitarian cost of such a policy change, mostly because of its ideological commitments. Thus, the critics picture the reform movement as a neoliberal scheme in which human subjectivity is brushed aside for the sake of efficiency, and all governance is left to technologically enhanced processes of self-regulation in the market. However, these critics might be deeply mistaken in their analogy between the new science envisaged by the reform movement and the neoliberal market—simply because their analyses are based solely on the commonality of some words (e.g., transparency, openness, and efficiency) with almost no regard to what they mean in the scientific reform context. What the critics miss, in our opinion, is that the reform movement can largely be seen as an attempt to redefine these “values.” As they stand, transparency, openness, and efficiency all have very specific meanings in the pre-reform context, and the reform movement can be said to have started a discussion about these meanings. “Transparency,” in the context of reform, is not exposing labor to the scrutiny of the market’s authority to maximize efficiency but a measure against fraud and questionable research practices that are incentivized by the neoliberal credit economy of the current academia. “Openness” is not a tool for mass data production and management using the tools of platform capitalism but a measure against the privatization of the outcomes, tools, and materials of publicly funded research by corporations and the prevention of the public from access to science and science-policy processes (Wilbanks & Topol, 2016). “Efficiency” is not the vision of increasing productivity (often at the expense of scientific quality and the well-being of laborers) but a concern with the alarming lack of credibility/quality in the scientific literature, which turns vast public resources and human labor into outputs that serve career advancement much more than the accumulation of scientific knowledge. The reformers understand from efficiency a reduction in the amount or proportion of scientific claims that lack sufficient theoretical, methodological, and evidential justification, which are consequently exceedingly difficult to interpret or uninformative (Button et al., 2013; Lakens & Evers, 2014). Second, the reformers view efficiency as a collective property of scientific inquiry in terms of increasing the level of coordination between scientific projects and pursuits so that we do not perpetuate contested, sterile kinds of literature but progress our scientific knowledge (however incrementally) through systematic and informative studies (see, e.g., Tiokhin, Panchanathan, et al., 2021; Uygun Tunç & Tunç, 2020; Yarkoni, 2022). Both aims are in conflict with the consequences of the neoliberal understanding of efficiency. The current conception of efficiency leads to stand-alone, novel, and bold claims that are publishable (although often insufficiently justified), and this serves to maximize the profits of corporate science publishers. The reformers’ new conception reduces this kind of efficiency, arguably for the benefit of science and the general public—so we can here see a clear difference. In arguing against the reformers’ allegedly neoliberal notion of efficiency, Peterson and Panofsky (2021, p. 352) very clearly (and ironically) illustrated the former sense of efficiency that is highly resonant with the neoliberal ethos and in tension with the aims and goals of the scientific reform:

Under this alternative theory of scientific efficiency, there is a natural process in which researchers produce many claims. Some may be flat wrong. Some may be right, yet hard to reproduce, or only narrowly correct and, therefore, be of limited use. However, some provide robust and exciting grounds to build upon and these become the shoulders on which future generations stand. . . . Reallocating resources to perform a rear-guard action of ensuring reproducibility reduces the funding that goes to producing new science (p. 352).

As it becomes clear from these (rather superficial) observations, the neoliberal ethos characterizes the scientific-reform movement much less than it does the very system of academic research that it endeavors to change. Krishna (2020) clearly described the sociopolitical outlook of contemporary academia amid profit-oriented privatization of science in terms of the abolition of the social contract between science and society and the erosion of the conception of scientific knowledge as a public good. The casualties of this transformation, for Krishna, are “open science as opposed to intellectual property rights, science for the public good as opposed to market good, peer review, and the prominence attached to open publications” (p. 11). The scientific-reform movement, on the other hand, effectively serves to restore the social contract between science and society through open data and material, open access, open peer review, and open educational resources that serve to make many elements of scientific knowledge accessible by the wider society.

## Maybe Not So Neoliberal: Team Science and Open Data Sharing

We explained earlier why we believe that the scientific-reform movement cannot be characterized by a single ideological stance and why the scholarly focus should be on particular policy proposals rather than the entire movement when investigating the sociopolitical backdrop of reform. Next, we do what we preach and examine the calls for collaborative or team science and open data sharing. One reason to select these examples is that they are widely popular among reformers, and another is that the critics of scientific reform pointed these proposals out as some obvious examples of reform being an appendage of neoliberalism. So, we think, an alternative interpretation of these policy proposals might be beneficial for the discussion.

### Team science

The campaign for team science advocates that various methodological problems that impede scientific progress can be tackled by cooperation among researchers and coordination of research projects. Collaborative research increases the scale of all kinds of resources devoted to particular research projects, from time allocation to the diversity of expertise and the complexity of technological infrastructure (Forscher et al., 2020). This wide-scale resource allocation can be undertaken with various methodological aims. For instance, in high-energy physics, a large-scale division of cognitive labor enables multiple cross-checking mechanisms for error detection and calibration of instruments, and validation mechanisms such as sister experiments (e.g., ATLAS and CMS at CERN; see Uygun Tunç, 2021). In the social, behavioral, and life sciences, a pertinent example is collaborative replication projects that utilize multisite replication studies to increase statistical power and check for the possible confounding effects of experimental settings, population characteristics, researcher bias, or sample selection (see Moshontz et al., 2018). Many Labs 2 (Klein et al., 2018), for instance, pooled together independent replication results to examine variation in effect sizes across samples and settings. As a science-policy proposal, the call for organizing scientific inquiry in the form of large research collaborations can be said to have a higher affinity with certain scientific values over others. Team science facilitates the use of resources to increase robustness, rigor, and reliability while penalizing novelty by limiting the number of individual research questions that can be investigated with the same resources. Another distinguishing feature of team science is that, unlike the individual-centered traditional research model, it allows for plurality in theoretical and methodological perspectives to be applied in tackling complex questions (Tebes et al., 2014), thereby increasing its potential affinity with scientific values such as diversity, pluralism, and perspectivism (Giere, 2010; Massimi & McCoy, 2020; Norgaard, 1989). Regarding broader sociopolitical values that shape the scientific ethos, team science promotes diversity in expertise and division of cognitive labor while diminishing the importance of eminence. A most concrete sign of this is the practice of consortium or group authorship (see, e.g., Fontanarosa et al., 2017), which implies substantial changes in how credit and responsibility are allocated in science. The high complexity of many contemporary research questions typically surpasses the expertise as well as the cognitive and physical capacities of individual researchers, which makes it increasingly difficult to investigate them within the neoliberal scientific culture and academic settings that primarily focus on individual eminence (see Tebes et al., 2014). Institutionally, large-scale collaborative projects cannot proliferate within an academic culture that values the quantity of first-author publications, novel hypotheses, and rapid results. On the basis of her observations of high-energy physics experiments, Knorr-Cetina (1999) described the new ethos demanded by collaborative research as (posttraditional) communitarianism, which is recognized by features such as collective ownership of scientific discovery, collective decision-making and responsibility, and the free flow of information. A communitarian ethos also cultivates quite different qualities in individuals than an individualist one. Within team-science contexts, specialized technical skills and field knowledge must be accompanied by social qualities of scientists that facilitate interpersonal cooperation, collective decision-making, and decentralized governance (see Koch & Jones, 2016). The current academic setting penalizes individual qualities that facilitate cooperation and rewards those that serve competition because the former do not directly contribute to measurable research outputs. For this reason, the call for team science also means a call for selecting qualities to reward not on an individual but on a collective level (Tiokhin et al., 2021) Tiokhin, Panchanathan, et al., 2021). So, unlike the critics (Callard, 2022; Mirowski, 2018; Morawski, 2019), we see little reason to associate team science with neoliberal ethos.

### Open data sharing

Open data sharing is a broad topic, but in the form which is defended by science reformers, its chief function is to enable other researchers to perform, reproducibility and replicability checks on published studies. It thus serves scientific quality control by facilitating first-order scientific criticism. Although critics raise the concern that mandating open data through science policy could lead to various sorts of harm (Levin & Leonelli, 2017; Peterson & Panofsky, 2021), we must bring attention to the other side of the coin: When there is no or limited access to data, trust in the scientific integrity, as well as the methodological rigor of the researcher, becomes mandatory. Because the critics prefer to underline that data sharing should be voluntary, a kind of courtesy or gift, we must add that such leeway also means increased vulnerability against problematic practices ranging from bias in data cleaning and selective reporting to outright data fabrication. In a system in which problematic research practices that serve to increase the publishability of studies are in effect incentivized, as is the case in many scientific fields today, this vulnerability could even result in the utter loss of the credibility of research outputs in the long run.

In addition to being motivated by concerns related to methodology and axiology, open data sharing also has a broader value dimension. Open data, like many other forms of openness, cultivates two Mertonian norms: communism, which prescribes collective ownership of scientific goods as opposed to secrecy, and organized skepticism, which prescribes critical scrutiny of scientific claims with respect to methodology and codes of scientific conduct (Merton, 1973; see also Vazire, 2018; Vicente-Saez & Martinez-Fuentes, 2018). Both of these values are jeopardized in the pre-reform academic context, in which the combination of high demand for constantly publishing novel and striking results with little demand for open data sharing undermines the capacity as well as the willingness of researchers to perform scientific criticism (Spellman, 2015).

## Conclusion

Because the reform movement has the potential to substantially reshape the nature of the scientific activity, a critical examination of its sociopolitical antecedents and consequences is deeply needed. Such a scholarly work would also be beneficial for the movement itself by broadening its reflective perspective. That being said, our examination of the published literature on reform criticism that associates the movement with the neoliberal status quo led us to conclude that we need a much more nuanced account of the sociopolitical underpinnings of reform. First, it is methodologically misguided to conceptualize the relationship between methodology and ideology as a direct and strong link. This is because models assuming causality, uniformity, or simplicity are higher-commitment than those assuming complexity and heterogeneity, and thus their choice should be justified. In this case, this would require offering a theoretically coherent mechanism by which methodology and ideology are directly linked, but none of the critics offers such a mechanism. Second, and related to the first point, critics tend to imagine the reform movement as a unitary entity that came together with a comprehensive consensus on axiology, science policy, and ideology. This is false because the movement features quite incompatible proclivities under its wing. Third, the haphazard connection that the critics made between neoliberalism and reform does not hold fast under closer scrutiny. The critics either selectively represent the arguments put forward by some names associated with the reform movement and neglect various other examples falsifying their position or misrepresent the movement by merely focusing on the lexical similarity of some of the values promoted by the science reformers with those cherished by neoliberal academia—while failing or refusing to see that a considerable portion of the science reformers actually strives to change the meaning of these words. To put things in perspective, the reform movement can be seen in part as an insurgency against old academia, particularly the shape it took after the neoliberal public policies of the 1980s. And we would like to believe that the critics voiced their misgivings because they saw an alarming tendency in the movement toward reproducing or at least reaching a compromise with that status quo. We should acknowledge that there is indeed such a risk. In the near future the methodology and science-policy changes envisioned by the reformers (e.g., preregistration, open data sharing) could be completely gamified, assimilated by the problematic status quo, lose their soul as well as rationale, and just become further ritual hurdles that researchers are expected to overcome in their competition for credit, awards, and promotions (see Claesen et al., 2021; Hardwicke et al., 2018)—not unlike what happened to Type 1 error control (i.e., *p* values). Yet sociopolitical analyses that overlook the historical context of the reform (in terms of the past it revolts against and the possible future states it might evolve into) cannot provide the movement with such a critical vision that it arguably seriously needs. What we need are much more nuanced accounts that recognize the major differences between various strands of the movement and diligently establish the associations between methodology, axiology, science policy, and ideology while showing respect to the particularities of individual cases. We hope this article contributes toward this goal and helps in the development of such well-grounded accounts in the future.

## Transparency

*Action Editor:* Laura A. King

*Editor:* Laura A. King

*Author Contributions*

D. Uygun Tunç and M. N. Tunç conceived, visualized, prepared, reviewed, and edited the manuscript. Z. B. Eper conceived and reviewed the manuscript. All of the authors approved the final manuscript for submission.

## Notes

1. Although the term “open science” predates the credibility crisis in psychological science and can be argued to cover much more ground, it is mostly merged with the scientific-reform movement.

2. Not all of the articles cited here are mainly about the relationship between open science and neoliberalism, and they were written by authors with substantially diversified understandings of science, except for making a similar assumption regarding the political outlook of the open-science reform movement. Relatedly, our criticism is not directed toward these articles in toto but to their pertinent parts. In this context, we also would like to state that we do not define a category such as “open-science critics” because many (if not most) of the “members” of the movement are also quite critical about some aspects of the movement, and many (if not most) of those who are conveniently called “the critics” are also part of the movement in our opinion. Therefore, we use the expression (i.e., the critics) only as shorthand for “authors who criticize open science on account of being neoliberal.”

3. The complexity of the interrelations between various domains (thus, the falsity of the holistic model of science) makes it highly unlikely for a phenomenon such as the scientific-reform movement to be a monolith, but the high heterogeneity of the open-science movement is also partly due to its history. We could possibly speak of a normatively unified phenomenon if open science were not a grassroots movement but a science-policy program governed and implemented from the top down by a central institutional body, such as a national research council. Moreover, it is not impossible that the open-science movement somehow evolves into a social organization with a unified sociopolitical group view in the future.
